# Reduced serum concentrations of 25-hydroxy vitamin D in children with autism: Relation to autoimmunity

**DOI:** 10.1186/1742-2094-9-201

**Published:** 2012-08-17

**Authors:** Gehan A Mostafa, Laila Y AL-Ayadhi

**Affiliations:** 1 Autism Research and Treatment Center, AL-Amodi Autism Research Chair, Department of Physiology, Faculty of Medicine, King Saud University, Riyadh, Saudi Arabia; 2 Department of Pediatrics, Faculty of Medicine, Ain Shams University, Cairo, Egypt; 3 9 Ahmed El-Samman Street off Makram Ebaid, Nasr City, Cairo, Egypt

**Keywords:** Anti-myelin-associated glycoprotein antibodies, Autism, Autoimmunity, Childhood autism rating scale, Vitamin D

## Abstract

**Background:**

Aside from the skeletal health affection, vitamin D deficiency has been implicated as a potential environmental factor triggering for some autoimmune disorders. Vitamin D might play a role in the regulation of the production of auto-antibodies. Immunomodulatory effects of vitamin D may act not only through modulation of T-helper cell function, but also through induction of CD4^+^CD25^high^ regulatory T-cells. We are the first to investigate the relationship between serum levels of 25-hydroxy vitamin D and anti-myelin-associated glycoprotein (anti-MAG) auto-antibodies in autistic children.

**Methods:**

Serum levels of 25-hydroxy vitamin D and anti-MAG auto-antibodies were measured in 50 autistic children, aged between 5 and 12 years, and 30 healthy-matched children. Serum 25-hydroxy vitamin D levels 10–30 ng/mL and < 10 ng/mL were defined as vitamin D insufficiency and deficiency, respectively.

**Results:**

Autistic children had significantly lower serum levels of 25-hydroxy vitamin D than healthy children (*P* < 0.001) with 40% and 48% being vitamin D deficient and insufficient, respectively. Serum 25-hydroxy vitamin D had significant negative correlations with Childhood Autism Rating Scale (*P* < 0.001). Increased levels of serum anti-MAG auto-antibodies were found in 70% of autistic patients. Serum 25-hydroxy vitamin D levels had significant negative correlations with serum levels of anti-MAG auto-antibodies (*P* < 0.001).

**Conclusions:**

Vitamin D deficiency was found in some autistic children and this deficiency may contribute to the induction of the production of serum anti-MAG auto-antibodies in these children. However, future studies looking at a potential role of vitamin D in the pathophysiology and treatment of autism are warranted.

## Background

Vitamin D is the common denominator of a group of sterols with a crucial role in calcium and phosphorus metabolism. The main source of vitamin D is the conversion of 7-dehydrocholesterol to pre-vitamin D3 in the skin, by means of solar ultraviolet B radiation, and a lesser amount of vitamin D is obtained from food. Vitamin D3 undergoes a 25-hydroxylation in the liver, with the resulting product, 25-hydroxy vitamin D or calcidiol, being the main circulating form of vitamin D. Therefor, 25-hydroxy vitamin D levels are used to determine the vitamin D status of a given individual [1,2]. The fully active form 1, 25 dihydroxy vitamin D3, is synthesized in the kidneys by the 25-hydroxy vitamin D-1a hydroxylase, an enzyme which is mainly induced by the parathyroid hormone (PTH). The main metabolic effect of 1, 25 dihydroxy vitamin D3, which is mediated through the interaction with vitamin D receptors, is promoting the intestinal absorption and renal resorption of calcium in order to increase its circulating levels. Deficient levels of vitamin D promote PTH synthesis that results in bone resorption. Long-lasting depletion of vitamin D causes rickets and osteomalacia, with skeletal deformities in children and bone pain and increased risk of fractures in adults, respectively [3].

A role for vitamin D in the regulation of immune function was first proposed after the identification of vitamin D receptors in lymphocytes. It has since been recognized that the active form of vitamin D has direct affects on naïve and activated helper T cells, regulatory T cells, activated B cells, and dendritic cells. There is a growing body of literature linking vitamin D to various immune-related conditions, including allergy and autoimmunity [4]. Recently, vitamin D deficiency has been implicated as a potential environmental factor triggering some autoimmune disorders, including multiple sclerosis (MS) and systemic lupus erythematosus (SLE) [5-7].

Autoimmunity may have a role in the pathogenesis of autism in a subgroup of patients. This may be indicated by the presence of brain-specific auto-antibodies in some autistic children [8-15]. There is also an increase in the frequency of autoimmune disorders among autistic families [16-22].

This study was the first to investigate the relationship between serum levels of 25-hydroxy vitamin D and anti-MAG auto-antibodies, as indicators of autoimmunity to brain tissue, in a group of autistic children.

## Methods

### Study population

This cross-sectional study was conducted on 50 children with autism. They were recruited from the Autism Research and Treatment Center, Faculty of Medicine, King Saud University, Riyadh, Saudi Arabia. Patients were fulfilling the criteria of the diagnosis of autism according to the fourth edition of the *Diagnostic and Statistical Manual of Mental Disorders*[23]. The autistic group comprised 39 boys and 11 girls. Their ages ranged between 5 and 12 years (mean ± SD = 8.24 ± 2.37 years). Patients who had associated neurological diseases (such as cerebral palsy and tuberous sclerosis) and metabolic disorders (for example, phenylketonuria) were excluded from the study. Also, autistic patients on casein-free diet were not included.

The control group comprised 30 age- and sex-matched apparently healthy children. They included twenty-four boys and six girls. They were the healthy older siblings of the healthy infants who attend the Well Baby Clinic, King Khalid University Hospital, Faculty of Medicine, King Saud University, Riyadh, Saudi Arabia for routine follow-up of their growth parameters. The control children were not related to the children with autism, and demonstrated no clinical findings suggestive of immunological or neuropsychiatric disorders. Their ages ranged between 5 and 12 years (mean ± SD = 8.63 ± 2.65 years).

All studied subjects had normal body weight (body mass index (BMI) was between the fifth and less than the 85th percentiles based on age and sex). In addition, all subjects were studied during summer (April to September) to avoid the effect of seasonal variation on serum 25 hydroxy vitamin D levels. Hours of sun exposure per week were recorded for all studied subjects. All studied children had not received calcium and/or vitamin D therapy in the past 6 months. In addition, none of them had a concomitant infection, photosensitivity, used photoprotection (such as broad-spectrum sunscreens) or treatment known to affect serum 25 hydroxy vitamin D levels (such as anti-epileptic drugs, corticosteroids, and other immunosuppressive drugs).

The local Ethical Committee of the Faculty of Medicine, King Saud University, Riyadh, Saudi Arabia, approved this study. In addition, an informed written consent of participation in the study was signed by the parents or the legal guardians of the studied subjects.

### Study measurements

#### Clinical evaluation of autistic patients

This was based on clinical history taking from caregivers, clinical examination, and neuropsychiatric assessment. In addition, the degree of the disease severity was assessed by using the Childhood Autism Rating Scale (CARS**)**[24] which rates the child on a scale from 1 to 4 in each of 15 areas (relating to people; emotional response; imitation; body use; object use; listening response; fear or nervousness; verbal communication; non-verbal communication; activity level; level and consistency of intellectual response; adaptation to change; visual response; taste, smell and touch response; and general impressions). According to the scale, children who have scored 30 to 36 have mild to moderate autism (*n* = 18), while those with scores ranging between 37 and 60 points have a severe degree of autism (*n* = 32).

#### Assay of serum 25-hydroxy vitamin D

This assay employs by enzyme-linked immunosorbent assay using a competitive protein binding assay kit for the measurement of 25-hydroxy vitamin D, which is based on the competition of 25-hydroxy vitamin D present in the sample with 25-hydroxy vitamin D tracer, for the binding pocket of vitamin D protein (VDBP, Gc-globulin). According to current recommendations, serum 25-hydroxy vitamin D levels < 30 and < 10 ng/mL were defined as vitamin D insufficiency and vitamin D deficiency, respectively, while levels > 30 ng/mL were defined as vitamin D sufficiency [25].

#### Assessment of serum anti-myelin associated glycoprotein (anti-MAG) antibodies

This assay employs the quantitative sandwich-type enzyme immunoassay (EIA) technique (Buhlmann Laboratories AG, Baselstrasse 55, CH-4124 Schonenbuch, Switzerland). Highly purified MAG from human brain has been precoated into a microtiter plate. Calibrators and patient sera were incubated for in the microtiter wells and any anti-MAG auto-antibodies present were bound by the immobilized human MAG. After washing away any unbound substances, horseradish peroxidase labeled antibodies against human IgM were added to the wells and incubated. After a wash step, the substrate solution containing tetramethylbenzidin was added. The color developed in proportion to the amount of anti-MAG antibodies bound in the initial step was stopped by adding an acidic stop solution. The intensity of the color absorbance was measured in a microtiter plate reader at a wavelength of 450 nm. To increase accuracy, all samples were analyzed twice in two independent experiments to assess the interassay variations and to ensure reproducibility of the observed results (*P* > 0.05). No significant cross-reactivity or interference was observed.

### Statistical analyses

The results were analyzed by the commercially available software package (Statview, Abacus concepts, Inc., Berkley, CA, USA). The parametric data were presented as mean and standard deviation (SD). In addition, non-parametric data were presented as median and interquartile range (IQR), which is the difference between the 75th and 25th percentiles. Students t-test was used for comparison of parametric data, while Mann–Whitney U-test was used for comparison of non-parametric data. Chi-square test was used for comparison between qualitative variables of the studied groups. Spearman’s rho correlation coefficient ‘r’ was used to determine the relationship between different variables. For all tests, a probability (*P*) of <0.05 was considered significant. Two SD above the mean value of serum anti-MAG auto-antibodies of healthy controls, 1983.45 Buhlmann titre unit (BTU), were considered to be elevated in autistic patients.

## Results

### Serum levels of 25-hydroxy vitamin D in autistic patients and healthy children

**Figure 1:**
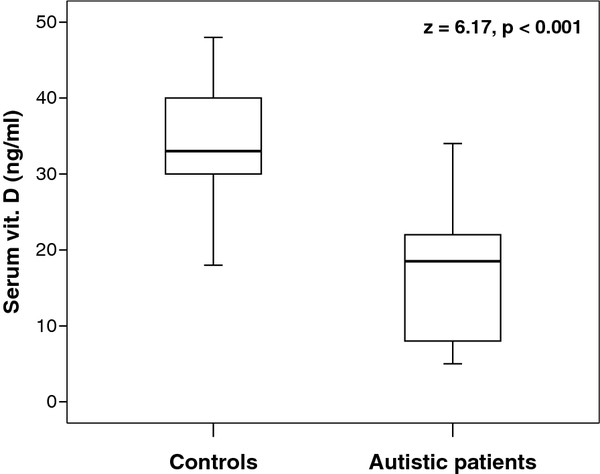
**Serum levels of 25-hydroxy vitamin D in the studied children.** The boxes enclose the interquartile range (IQR), which are between the 25th and 75th percentiles. The horizontal line inside the box represents the median and the whiskers represent the non-outlier (between 1.5 and 3 IQR) or extreme (more than 3 IQR) maximum and minimum values of serum 25-hydroxy vitamin D.

Autistic children had significantly lower serum levels of 25-hydroxy vitamin D (median (IQR) = 18.5 (14) ng/mL) than healthy children (median (IQR) = 33 (11) ng/mL), *P* < 0.001 (Figure 1), with 40% and 48% being vitamin D deficient and insufficient, respectively. None of the healthy children was vitamin D deficient, but 20% were vitamin D insufficient (Table 1).

**Table 1:** Basic clinical data and vitamin D status of the studied children

**Clinical data**	**Autistic children ( *n* = 50)**	**Control children ( *n* = 30)**
Age ( years)	8.24 ± 2.37	8.63 ± 2.65
Sex (male/female)	39/11	24/6
The duration of sun exposure (hours/week)	6.48 ± 1.29	6.23 ± 1.41
Vitamin D deficiency (<10 ng/mL)	40% (20/50)	0
Vitamin D insufficiency (10 to 30 ng/mL)	48% (24/50)	20% (6/30)
Vitamin D sufficiency (>30 ng/mL)	12% (6/50)	80% (24/30)

Serum 25-hydroxy vitamin D had significant negative correlations with CARS (r = −0.84, *P* < 0.001). Although patients with severe autism had lower serum 25-hydroxy vitamin D than children with mild to moderate autism, this difference did not reach statistical significance, *P* = 0.06 (Table 2). Serum 25-hydroxy vitamin D had no significant correlations with the age of autistic children (*P* = 0.83). In addition, there was no significant difference between male and female autistic children in serum levels of 25-hydroxy vitamin D (*P* > 0.05).

**Table 2:** Serum levels of 25-hydroxy vitamin D and anti-MAG auto-antibodies in relation to the severity of autism

	**Patients with mild to moderate autism ( *n* = 18)**	**Patients with severe autism ( *n* = 32)**	**Z/T ( *P* value)**
Serum 25-hydroxy vitamin D (ng/mL) (median (IQR))	20.5 (16)	14.5 (15)	1.82 (0.06)
Serum anti-MAG (BTU) (mean ± SD)	1827.5 ± 349.9	2525.5 ± 529.6	5.6 (<0.001)

Anti-MAG, anti-myelin-associated glycoprotein antibodies.

There was no significant difference between autistic children and healthy children in the duration of sun exposure/week (*P* = 0.49). In addition, serum 25-hydroxy vitamin D had no significant correlations with the duration of sun exposure/week (*P* = 0.96).

### Serum levels of anti-MAG auto-antibodies in autistic patients and healthy children

**Figure 2:**
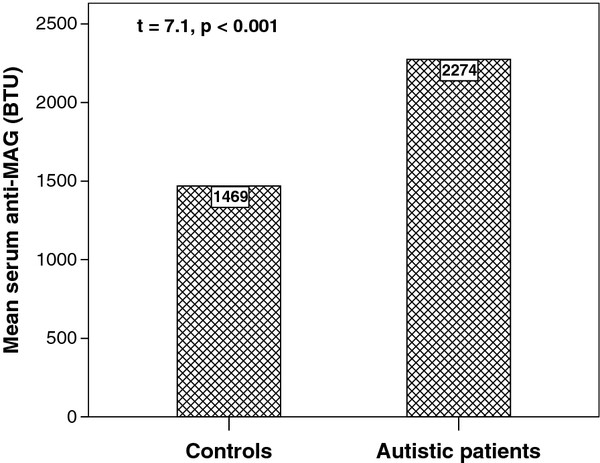
**Serum levels of anti-MAG auto-antibodies in the studied children.** Anti-MAG, anti-myelin-associated glycoprotein antibodies; BTU, Buhlmann titre unit.

Autistic children had significantly higher serum levels of anti-MAG auto-antibodies (2274.22 ± 578.32 BTU) than healthy children (1468.57 ± 302.89 BTU), *P* < 0.001 (Figure 2). According to the highest cut-off values of serum anti-MAG auto-antibodies, increased levels were found in 70% of autistic patients.

Patients with severe autism had significantly higher serum anti-MAG auto-antibodies than children with mild to moderate autism, *P* < 0.001 (Table 2). In addition, children with severe autism had significantly higher frequency of seropositivity of anti-MAG auto-antibodies (90.6%) than autistic patients with mild to moderate autism (33.3%), *P* < 0.001. Also, serum anti-MAG auto-antibodies had significant positive correlations with CARS (r = 0.81, *P* < 0.001). In contrast, serum anti-MAG auto-antibodies had no significant correlations with the age of autistic children (*P* = 0.5).

### The relationship between serum levels of 25-hydroxy vitamin D and anti-MAG auto-antibodies in autistic children

**Figure 3:**
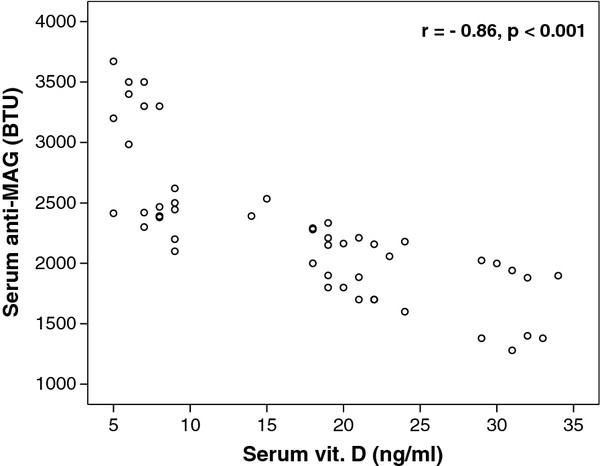
**Negative correlations between serum levels of 25-hydroxy vitamin D and anti-MAG auto-antibodies in children with autism.** Anti-MAG, anti-myelin-associated glycoprotein antibodies; BTU, Buhlmann titre unit.

Serum 25-hydroxy vitamin D levels had significant negative correlations with serum levels of anti-MAG auto-antibodies (*P* < 0.001), Figure 3.

## Discussion

Immune abnormalities, mainly autoimmunity to brain tissue, may have a pathogenic role in autism [26-29]. Recently, vitamin D deficiency has been implicated as a potential environmental factor triggering some autoimmune disorders [5,6].

In the present study, autistic children had significantly lower serum levels of 25-hydroxy vitamin D than healthy children, *P* < 0.001, with 40% and 48% being vitamin D deficient and insufficient, respectively.

Vitamin D deficiency is being associated with a number of psychiatric conditions with a developmental basis, such as autism and schizophrenia. Vitamin D deficiency in early life affects neuronal differentiation, axonal connectivity, dopamine ontogeny, and brain structure and function [30]. Some investigators reported reduced serum 25-hydrox vitamin D in autistic children. These studies could classify them as being ‘vitamin D inadequate’, which lends support to the hypothesis that autism is a vitamin D deficiency disorder [31,32].

Worldwide, the rate of autism has been steadily rising. There are several environmental factors in concert with genetic susceptibilities that are contributing to this rise. Genetic polymorphisms of cytochrome P450 enzymes have also been linked to autism, specifically CYP27B1 that is essential for proper vitamin D metabolism. Three novel structural variants of the vitamin D receptor were reported in psychiatric disease [33]. Further study is required to clarify their role, if any, in autism.

In the present work, serum 25-hydroxy vitamin D levels had significant negative correlations with CARS (*P* < 0.001) which signifies the possible link between the extent of vitamin D deficiency and the degree of the severity of autism. Vitamin D receptors and vitamin D metabolizing enzymes are present in CNS. Calcitriol, the active vitamin D, affects numerous neurotransmitters and neurotrophic factors, relevant for mental disorders [34]. In addition, vitamin D plays important roles in repairing DNA damage and protecting against oxidative stress which is a key cause of DNA damage. Thus, vitamin D deficiency may contribute to higher mutation rates and impaired repair of DNA [35] as a result of increased oxidative stress which was reported in some autistic children [22]. In addition, Calcitriol may down-regulate the production of inflammatory cytokines in the brain that have been suggested to be associated with autism [36].

Maternal vitamin D deficiency may be a risk factor for autism, possibly by affecting fetal brain development as well as by affecting maternal immune system status during pregnancy [37,38]. Autism was more common among children of the mothers who took antiepileptic drugs as these drugs are one of the few classes of drugs that consistently and significantly interfere with vitamin D metabolism [39]. Unfortunately, the tiny 10 μg (400 IU) dose in prenatal vitamins is virtually irrelevant in preventing gestational vitamin D deficiency [40]. For this reason, in 2007, the Canadian Paediatric Society recommended 2000 IU/day, or more, to prevent gestational vitamin D deficiency [25].

The current study revealed no significant difference between autistic children and healthy children in the duration of sun exposure/week (*P* = 0.49). In addition, serum 25-hydroxy vitamin D had no significant correlations with the duration of sun exposure/week in children with autism (*P* = 0.96). In addition, all subjects were studied during summer (April to September) to avoid the effect of seasonal variation on serum 25 hydroxy vitamin D levels. Thus, the results of our study did not signify the reduced duration of sun exposure as a causal factor of 25 hydroxy vitamin D deficiency in autistic children.

In this study, autistic children had significantly higher serum levels of anti-MAG auto-antibodies than healthy children, *P* < 0.001. Increased serum levels of anti-MAG auto-antibodies were found in 70% of autistic patients. A previous study conducted on 32 Egyptian children, aged between 3 and 8 years, reported anti-MAG seropositivity in 62.5% of autistic children [10]. MAG is a minor myelin protein that is located in the most periaxonal oligodendrocytes processes. It controls neurofilament phosphorylation and hence, controls the axon caliber which is crucial for efficient impulse transmission. In contrast to mature CNS, MAG promotes regeneration of young neurons [41]. Circulating anti-MAG antibodies may play an etiopathogenic role in some autoimmune disorders as autoimmune chronic demyelinating neuropathy [42]. Autoimmune reaction to neurons might be initiated by environment triggers resulting in the release of neuronal antigens, which through the activation of inflammatory cells, may result in autoimmune reactions in genetically susceptible individuals [26,27].

In the present work, patients with severe autism had significantly higher serum anti-MAG auto-antibodies than children with mild to moderate autism, *P* < 0.001. Also, serum anti-MAG auto-antibodies had significant positive correlations with CARS (*P* < 0.001). These results may indicate that the extent of the elevation of serum anti-MAG auto-antibodies was possibly linked to the degree of the disease severity assessed by CARS. Thus, anti-MAG auto-antibodies might be playing a role in the pathogenesis of brain damage, the extent of which may determine the clinical severity of autism.

Vitamin D deficiency has been implicated as a potential environmental factor triggering some autoimmune disorders suggesting that vitamin D might play a role in regulating auto-antibody production [5,6]. In this study, we have tried to find a possible link between the reduced serum levels of 25-hydroxy vitamin D and the elevated serum levels of anti-MAG auto-antibodies in autism. Serum 25-hydroxy vitamin D levels had significant negative correlations with serum levels of anti-MAG auto-antibodies (*P* < 0.001). We could not trace data in the literature concerning the possible contributing role of 25-hydroxy vitamin D deficiency in the induction of the production of brain-specific auto-antibodies in some autistic children to compare our results. We are the first to study such a relationship.

In animal models, an increased Th2 response to vitamin D has been accompanied by increase in anti-inflammatory IL-10 response [43]. Immunomodulatory effect of vitamin D may act not only through modulation of helper T cell function, but also through induction of CD4^+^CD25^high^ regulatory T-cells (Tregs) [44]. CD4^+^CD25^high^ regulatory T-cells play an important role in the establishment of the immunological self tolerance and thereby, preventing autoimmunity [45]. Tregs can suppress Th17 cells, the key players in the pathogenesis of autoimmune disorders, and autoimmunity [46]. A recent study reported deficiency of Tregs in 73.3% of autistic children [21]. Thus, vitamin D deficiency may be a contributing factor to the production of brain-specific auto-antibodies as a result of deficiency of Tregs in some autistic children.

The results of this study may indicate that 25-hydroxy vitamin D deficiency may be a possible contributing factor to the increased frequency of serum anti-MAG auto-antibodies in some autistic children. However, this is an initial report that warrants further research to determine the possible link between 25-hydroxy vitamin D deficiency and the increased frequency of brain-specific auto-antibodies in some children with autism.

The vitamin D theory of autism does not diminish genetic contributions to autism occurrence. Indeed, without the genetic tendency for autism, severe maternal or early childhood vitamin D deficiency may cause bone abnormalities with no evidence autism. Maternal and early childhood vitamin D deficiency may allow the genetic tendency for autism to express itself. If this theory is true, the path towards effective prevention and perhaps a treatment is so simple, so safe, so inexpensive, so readily available and so easy [47]. Basic, genetic, and epidemiological studies indicate a potential role of vitamin D in the prevention of autoimmune diseases, but randomized and controlled trials are necessary to establish the clinical efficacy of vitamin D supplementation in ill or at-risk subjects [48]. Three treatment modalities exist for vitamin D deficiency which include; sunlight, artificial ultraviolet B radiation, and vitamin D3 supplementation. Treatment of vitamin D deficiency in patients with 2,000 to 7,000 IU vitamin D per day should be sufficient to maintain year-round levels between 40 and 70 ng/mL [49]. Children with chronic illnesses such as autism, diabetes, and/or frequent infections should be supplemented with higher doses of sunshine or vitamin D3 [47].

## Conclusions

Vitamin D deficiency was found in some autistic children and this deficiency may contribute to the induction of the production of serum anti-MAG auto-antibodies in these children. However, future studies looking at a potential role of vitamin D in the pathophysiology and treatment of autism are warranted.

## Abbreviations

Anti-MAG, Anti-myelin-associated glycoprotein; CARS, Childhood Autism Rating Scale; IQR, Interquartile range; MS, Multiple sclerosis; PTH, Parathyroid hormone; SLE, Systemic lupus erythematosus; Th, T-helper; Tregs, CD4+CD25high regulatory T-cells.

## Competing interests

The authors declare that they have no competing interests.

## Authors’ contributions

Both authors designed, performed and wrote the research. In addition, both authors read and approved the final manuscript.
